# Exogenous Interleukin-33 Contributes to Protective Immunity via Cytotoxic T-Cell Priming against Mucosal Influenza Viral Infection

**DOI:** 10.3390/v11090840

**Published:** 2019-09-10

**Authors:** Chae Won Kim, Hye Jee Yoo, Jang Hyun Park, Ji Eun Oh, Heung Kyu Lee

**Affiliations:** 1 Biomedical Science and Engineering Interdisciplinary Program, Korea Advanced Institute of Science and Technology (KAIST), Daejeon 34141, Korea; 2 Graduate School of Medical Science and Engineering, KAIST, Daejeon 34141, Korea; 3 KAIST Institute for Health Science and Technology, KAIST, Daejeon 34141, Korea

**Keywords:** IL-33, influenza virus, antiviral immunity

## Abstract

Influenza is an infectious respiratory illness caused by the influenza virus. Though vaccines against influenza exist, they have limited efficacy. To additionally develop effective treatments, there is a need to study the mechanisms of host defenses from influenza viral infections. To date, the mechanism by which interleukin (IL)-33 modulates the antiviral immune response post-influenza infection is unclear. In this study, we demonstrate that exogenous IL-33 enhanced antiviral protection against influenza virus infection. Exogenous IL-33 induced the recruitment of dendritic cells, increased the secretion of pro-inflammatory cytokine IL-12, and promoted cytotoxic T-cell responses in the local microenvironment. Thus, our findings suggest a role of exogenous IL-33 in the antiviral immune response against influenza infection.

## 1. Introduction

Influenza is an acute infection of the respiratory tract that is caused by influenza viruses circulating all over the world. This disease is a public health problem, and the number of annual deaths associated with influenza is estimated to range from 290,000 to 650,000 worldwide [1]. Because adults over 65 years and children under four years of age are especially susceptible to influenza infection, vaccine administration against influenza is recommended in these populations. This vaccination can lead to decreases in incidence rate and severity [2]. However, the efficacy of vaccines remains low, as the virus can evolve with antigenic drifts and shifts, and it can be difficult to target and predict modified antigens [3,4,5]. Thus, the studying of antiviral immune responses against influenza infection is still needed to develop additional therapies for this disease.

Interleukin (IL)-33 is an alarmin that is released from damaged cells, especially epithelial and endothelial cells [6]. It is also released in the lung during influenza virus infection [7]. IL-33 can induce type 2 immune responses, such as allergic inflammation and asthma [8]. However, recent studies have shown that T helper 1 cells, cytotoxic T-cells, and regulatory T-cells also express IL-33 receptor ST2 under certain circumstances, such as bacterial or viral infections. As a result, IL-33 may affect the differentiation and function of these cells [9,10,11]. Thus, IL-33 plays a role in not only type 2 immunity but also in type 1 immunity against bacterial and viral infections.

The role of IL-33 in viral infections is complex. During a systemic infection with lymphocytic choriomeningitis virus, IL-33 promotes the expansion of viral-specific CD8^+^ T-cells, thus leading to an enhanced protection against viral infection [10]. However, during mucosal infection with herpes simplex virus type 2, the systemic administration of IL-33 increases susceptibility to viral infection, resulting in severe mortality and morbidity [12]. Though there have been some studies on the role of IL-33 in lung tissue damage or recovery [13,14], whether IL-33 enhances or exacerbates antiviral immunity during influenza infection remains unclear.

In this study, we show that exogenous IL-33 affected mortality against influenza infection by increasing dendritic cell (DC) recruitment, pro-inflammatory cytokine secretion, and cytotoxic T-cell responses in a local microenvironment. Overall, these results elucidated a role of IL-33 in antiviral immunity against the influenza virus, suggesting that IL-33 enhances protective immune responses against influenza virus infections.

## 2. Materials and Methods

### 2.1. Mice

Male C57BL/6 specific-pathogen-free mice (6–8 weeks old) were purchased from DBL Co., Ltd. (Chungbuk, Korea). IL-33^−/−^ mice were provided by the RIKEN Center for Developmental Biology [15] and were maintained in a specific facility at the Korea Advanced Institute of Science and Technology (KAIST). Male IL-33^−/−^ mice (8–15 weeks old) were used for experiments. All experiments followed the guidelines of the Institutional Animal Care and Use Committee of KAIST (KA2013-55, 24 October 2013).

### 2.2. Recombinant IL-33 Treatment and Influenza Virus Infection In Vivo

Mice were anesthetized via isoflurane inhalation and then injected intranasally with 0.5 μg of recombinant IL-33 (rIL-33; BioLegend, San Diego, CA, USA) in 20 μL phosphate-buffered saline (PBS) daily for 5 days prior to virus infection.

Influenza virus strain (strain A/Puerto Rico/8/1934 H1N1, PR8) was provided by A. Iwasaki (Yale University, New Haven, CT, USA). For intranasal virus infections, mice were injected intraperitoneally with ketamine (100 mg/kg) and xylazine (5.83 mg/kg) for anesthesia, followed by intranasal injections of 50 pfu PR8 in 20 μL PBS. After infection, mice were monitored for survival and body weight until body weight loss reached 75% of their initial weight.

### 2.3. Viral Titration and Measurement of Cytokines in Bronchoalveolar Lavage (BAL) Fluids

For viral titration and cytokine measurements, bronchoalveolar lavage (BAL) fluids from infected mice were collected at the indicated time points by washing with 1 ml of PBS [16].

The viral titers were determined by a standard PR8 plaque assay on Madin–Darby canine kidney (MDCK) cells, as previously reported [17]. Briefly, MDCK cells (1 × 10^6^ cells/well) were cultured in a 6-well plate and incubated in Dulbecco’s Modified Eagle Medium (DMEM) with 10% fetal bovine serum (FBS) and 1% penicillin/streptomycin (Welgene, Daegu, Korea) overnight at 37 °C. After confluency, culture media were removed, and serially-diluted BAL fluids (200 μL) were added to the wells. Cells were then incubated at 37 °C for 1 h with shaking every 20 min, after which they were washed twice with PBS. Afterwards, cells were incubated with 2 ml of a 0.6% agarose medium per well at 37 °C for 72 h. The number of plaques was counted.

The levels of IL-6, IL-12p40, and IL-33 in BAL fluids were measured by an enzyme-linked immunosorbent assay (ELISA; BD Bioscience, San Jose, CA, USA), according to the manufacturer’s protocol.

### 2.4. In Vitro Stimulation of Bone Marrow-Derived Dendritic Cells (BMDCs)

Bone marrow was isolated from C57BL/6 mice, and the isolated cells were differentiated into DCs according to a previously described protocol, with slight modifications [18]. These cells were seeded in a 24-well plate in RPMI 1640 (Welgene) with a 5% granulocyte-macrophage colony-stimulating factor (GM-CSF) medium. The medium was replaced every 2 days, and bone marrow-derived dendritic cells (BMDCs) were harvested at day 6.

BMDCs (2 × 10^5^ cells/well) were seeded in a 96-well plate and stimulated with 1, 10, or 100 ng/mL of rIL-33 at 12 h prior to infection with the PR8 virus (1, 10, or 50 multiplicity of infections [MOIs]). At 24 h post-stimulation or 48 h post-infection, the cells and the culture medium were harvested. The production of IL-12p40 in the culture medium was measured by ELISA (BD Bioscience), according to the manufacturer’s protocol. The expression of surface markers, such as CD11c-FITC (N418, Biolegend), CD86-PE (GL1, BD Bioscience), and MHCII-PerCP-cy5.5 (M5/114.15.2, BD Bioscience), in harvested BMDCs were analyzed by flow cytometry.

### 2.5. Isolation of Single Cells from Lungs

Immune cells were isolated from lungs using previously described methods [19]. Briefly, mice were euthanized with CO_2_ gas. Lung tissues were harvested and digested for 30 min at 37 °C in 1% FBS in DMEM with DNase I (Roche, Basel, Switzerland) and collagenase IV (Worthington Biochemical Corporation, Lakewood, NJ, USA). Digested tissues were centrifuged at 1500 rpm for 5 min at 4 °C, resuspended with 5 mL of Hank’s Balanced Salt Solution (Welgene) with 5 mM EDTA and 5% FBS, and incubated for 5 min at 37 °C. Then, single cells were suspended using a 70 µm cell strainer and plunger. These cells were applied to Percoll (GE Healthcare Life Sciences, Marlborough, MA, USA) density gradient centrifugation. After washing with PBS, harvested cells were treated with an ammonium–chloride–potassium lysis buffer to remove red blood cells.

### 2.6. Flow Cytometry

Single cells were stained with the following antibodies: B220-FITC (RA3-6B2, BioLegend), CD11b-FITC (M1/70, Tonbo), CD11c-FITC (N418, BioLegend), CD3-FITC (17A2, BioLegend), FcεRI-FITC (MAR-1, eBioscience), Ly6G-FITC (1-A8, BioLegend), NK1.1-FITC (PK136, BD Bioscience), ST2-PE (DIH9, BioLegend), MHCII-PerCP-Cy5.5 (M5/114.15.2, eBioscience), Sca-1-PerCP-Cy5.5 (D7, eBioscience), CD11c-PE-Cy7 (N418, BioLegend), CD45.2-AF700 (104, BioLegend), SiglecF-V421 (E50-2440, BD Bioscience), CD11b-V500 (M1/70, BD Bioscience), Propidium Iodide (PI) Staining Solution (eBioscience), and CD16/32 (2.4G2, BD Bioscience). Type 2 innate lymphoid cells (ILC2s), DCs, eosinophils, and alveolar macrophages were analyzed by BD LSRFortessa. These cells were first gated for PI-negative cells to exclude all dead cells. Forward scatter (FSC-A) strategies were used for doublet discrimination from single cells. Additionally, cells were gated for CD45.2-positive and lineage-negative (CD3, B220, NK1.1, CD11b, FcεRI, and CD11c) ILC2 cells. ILC2 cells were finally gated for Sca-1- and ST2-positive cells. The other cells were gated for Ly6G and CD11b double-negative cells to ensure neutrophil exclusion. Finally, cells were gated for MHCII and CD11c double-positive DCs as well as DC-negative and Siglec-F-positive alveolar macrophages and eosinophils. Then, alveolar macrophages were finally gated with CD11c^hi^ CD11b^int^, and eosinophils were gated with CD11c^int^ CD11b^hi^. The analysis of immune cells was performed according to previously described methods [20].

For pentamer staining, single cells were stained with CD3-FITC (17A2, Biolegend), NP_366–374_ Pentamer-PE (Proimmune), CD44-PerCP-Cy5.5 (IM7, Tonbo), and CD8α-APC (53-6.7, BD Bioscience). For interferon (IFN)-γ intracellular staining, single cells were stimulated ex vivo for 5 h with 10 μg/mL of NP_366–374_ (Peptron) or 50 ng/mL phorbol 12-myristate 13-acetate (PMA, Sigma, St. Louis, MO, USA) in addition to 1 μg/mL ionomycin (Sigma) and 0.134 μL protein transport inhibitor containing monensin (Golgistop, BD Bioscience), according to the manufacturer’s protocol. Then, stimulated cells were stained with CD4-FITC (GK1.5, Biolegend), CD8α-PE (53-6.7, Biolegend), CD44-PerCP-Cy5.5 (IM7, Tonbo), and CD16/32 (2.4G2, BD Bioscience). The stained cells were fixed and permeabilized by a fixation/permeabilization buffer (BD Bioscience) for 15 min followed by staining with IFN-γ-APC (XMG1.2, Biolegend) or Rat IgG1 κ-isotype control-APC (RTK2017, Biolegend). After pentamer and intracellular staining, these cells were analyzed by a BD FACSCalibur.

### 2.7. Statistical Analysis

All data are expressed as mean ± SEM. Differences in survival were analyzed using the log-rank test. Differences between groups were evaluated by unpaired, two-tailed Student’s *t* tests. Statistical analyses were performed using GraphPad Prism software (GraphPad). *p* < 0.05 was considered to be statistically significant.

## 3. Results

### 3.1. Exogenous IL-33, but Not Endogenous IL-33, Enhances Protective Immunity against Influenza Infection

**Figure 1:**
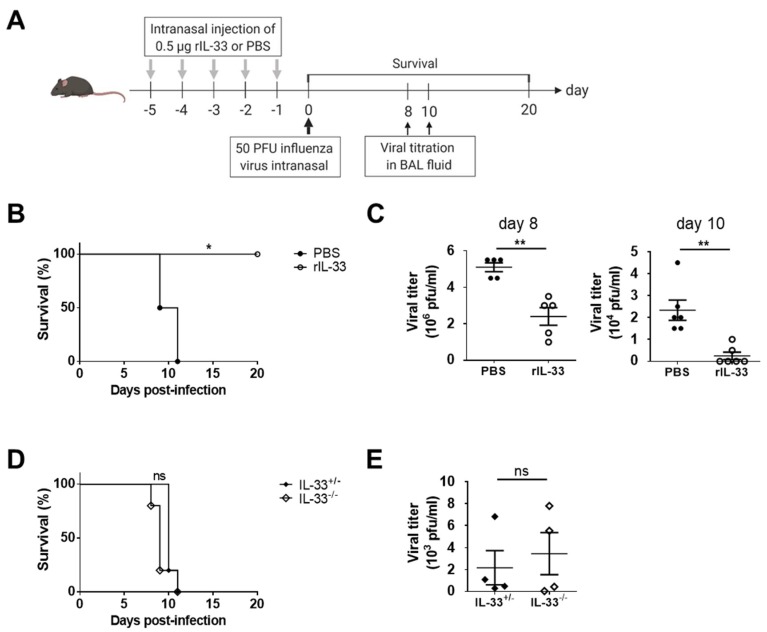
Exogenous interleukin (IL)-33, but not endogenous IL-33, improved the survival of mice infected with PR8 influenza virus. (**A**) C57BL/6 mice were injected intranasally with 0.5 μg of rIL-33 or phosphate-buffered saline (PBS) daily for five days and infected with 50 PFU of PR8 influenza virus. (**B**) Survival was monitored for 20 days post-infection. (**C**) At the indicated days post-infection, PR8 viral titers in BAL fluids were measured on Madin–Darby canine kidney (MDCK) cells. (**D**,**E**) IL-33^+/−^ and IL-33^−/−^ mice were intranasally infected with 50 PFU of PR8 influenza virus, and (**D**) survival was monitored for 10 days post-infection. (**E**) At eight days post-infection, PR8 viral titers in BAL fluids were measured in MDCK cells. * *p* < 0.05; ** *p* < 0.01; ns, not significant. BAL: Bronchoalveolar lavage.

To determine the effect of exogenous IL-33 on antiviral immunity against influenza infection, we examined the results of intranasal PR8 infection following daily intranasal injections of rIL-33 for five days (Figure 1A). PBS-treated mice died within two weeks. However, all mice that were injected with rIL-33 survived (Figure 1B). There was a tendency for viral titers to decrease in rIL-33-treated mice eight days post-infection, and the viral loads of most rIL-33-treated mice were not detected 10 days post-infection (Figure 1C).

An infection with a large amount (about 10^6^ plaque forming unit (PFU) per mouse) of influenza virus has been shown to induce IL-33 secretion [7]. However, in this study, the secretion of IL-33 was less than that of other inflammatory cytokines, including IL-6 and IL-12p40 (Figure S1). To determine the effect of endogenous IL-33 on antiviral immunity against influenza infection, we examined the outcomes of intranasal PR8 infection in IL-33-deficient mice. There was no significant difference in survival between IL-33^+/−^ and IL-33^−/−^ mice post-infection (Figure 1D). Additionally, viral titers from BAL fluids were not significantly different between groups (Figure 1E).

These results suggest that exogenous IL-33, but not endogenous IL-33, enhances the protective effect of antiviral immunity against influenza infection.

### 3.2. Exogenous IL-33 Induces the Recruitment of Dendritic Cells into the Lung

**Figure 2:**
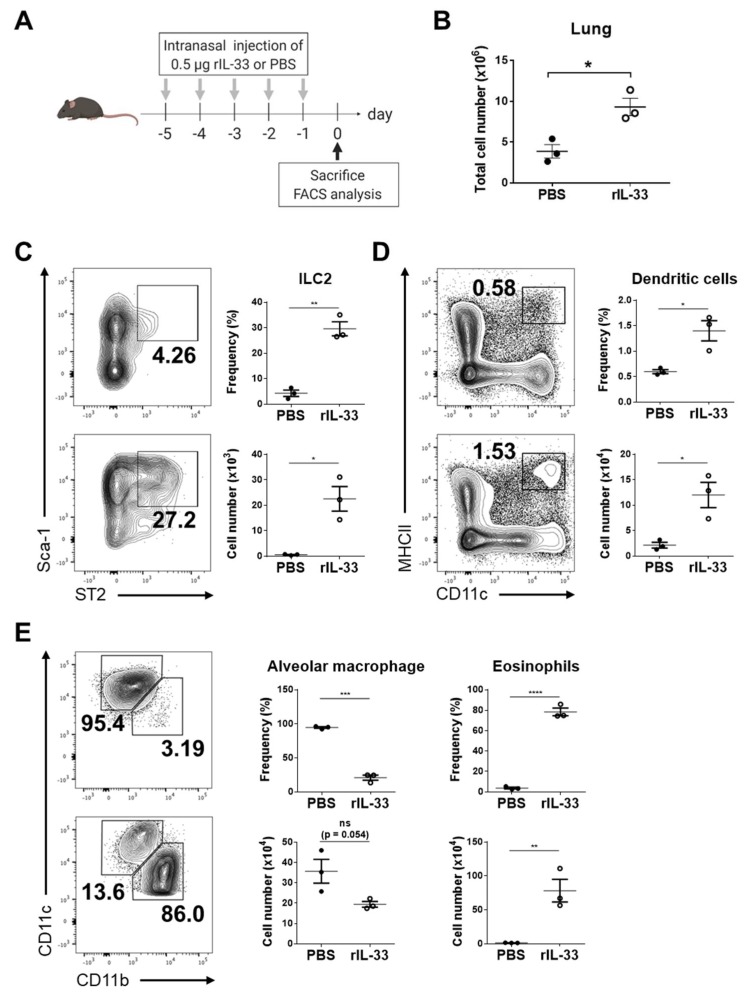
Exogenous IL-33 affected immune cells in the lung. (**A**) C57BL/6 mice were injected intranasally with 0.5 μg rIL-33 or PBS daily for five days, and lungs were collected for analyses of immune cell populations. (**B**) The numbers of cells isolated from the lungs were counted. The frequency and number of lineage-specific (**C**) Sca-1^+^ ST2^+^ type 2 innate lymphoid cells (ILC2s), (**D**) CD11c^+^ MHCII^+^ dendritic cell (DCs), (**E**) Siglec-F^+^ CD11c^hi^ CD11b^int^ alveolar macrophages, and Siglec-F^+^ CD11c^int^ CD11b^hi^ eosinophils were analyzed by flow cytometry. * *p* < 0.05; ** *p* < 0.01; *** *p* < 0.001; **** *p* < 0.0001; ns, not significant.

IL-33 is a ligand for the ST2 receptor, which is expressed on several immune cells, and induces type 2 immune responses. In particular, ILC2s are known as early responders of IL-33 with secretion of IL-5 and IL-13, and eosinophils are another well-known responder of IL-33 in allergic inflammation [8,9]. Thus, we first confirmed whether exogenous IL-33 increases ILC2s and eosinophil recruitment into the lungs (Figure 2A). The number of isolated cells from lungs of exogenous IL-33-treated mice was significantly higher than those of PBS-treated mice (Figure 2B). As expected, the frequencies and numbers of ILC2 and eosinophils significantly increased with exogenous IL-33 treatment (Figure 2C,E). In addition, we examined the frequencies and numbers of antigen-presenting cells, including DCs and alveolar macrophages, which are able to activate CD8^+^ T-cell responses against influenza infection. The frequency and number of DCs were significantly increased with exogenous IL-33 (Figure 2D), whereas the frequency and number of alveolar macrophages were decreased (Figure 2E). These results indicate that exogenous IL-33 recruits ILC2s, eosinophils, and DCs into lungs. Furthermore, alveolar macrophages were not affected.

### 3.3. Exogenous IL-33 Enhances IL-12p40 Secretion and DC Maturation

**Figure 3:**
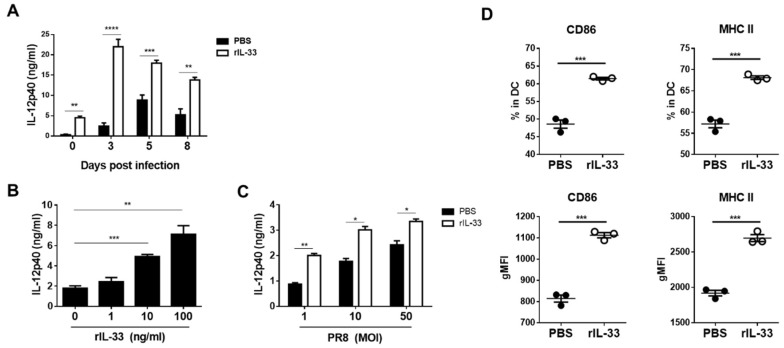
Exogenous IL-33 enhanced IL-12p40 secretion in BAL fluids, bone marrow-derived dendritic cell (BMDC) culture media, and BMDC maturation. (**A**) C57BL/6 mice were injected intranasally with 0.5 μg of rIL-33 or PBS daily for five days and then infected with 50 PFU of PR8 influenza virus. At the indicated days post-infection, BAL fluids were collected, and IL12p40 levels were measured by an enzyme-linked immunosorbent assay (ELISA). (**B**) BMDCs were stimulated with the indicated concentrations of rIL-33. At 24 h post-stimulation, IL-12p40 levels in culture media were measured by ELISA. (**C**) BMDCs were infected with 1, 10, and 50 multiplicity of infection (MOI) PR8 virus with 10 ng/mL of rIL-33 or PBS. At 24 h post-infection, IL-12p40 levels in culture media were measured by ELISA. (**D**) BMDCs were stimulated with 10 ng/ml of rIL-33, and the expression of CD86 and MHCII in BMDCs was assessed by flow cytometry at 24 h post-stimulation. * *p* < 0.05; ** *p* < 0.01; *** *p* < 0.001; **** *p* < 0.0001.

In influenza virus infections, IL-12 contributes to the induction of the early innate immune response [21,22]. IL-12 also inhibits apoptosis and promotes the production of IFN-γ by CD8^+^ T-cells [23]. Thus, we examined the levels of IL-12p40 in BAL fluids harvested from lungs at zero, three, five, and eight days post-infection. Despite pre-infection (day zero), IL-12p40 level in BAL fluids with exogenous IL-33 injection were higher than those with PBS. On other post-infection days, higher levels of IL-12p40 were measured in BAL fluids with exogenous IL-33 injection than PBS (Figure 3A).

The main sources of IL-12 are professional antigen-presenting cells, such as macrophages and DCs [24]. Our data show that exogenous IL-33 induced the recruitment of DCs but not alveolar macrophages. To confirm whether DCs secrete IL-12 in response to IL-33, we generated BMDCs and treated the cells with different concentrations of rIL-33. At 24 h after treatment, the levels of IL-12p40 in cell culture media were measured. The results show that secretion of IL-12p40 was enhanced as the concentration of rIL-33 was increased (Figure 3B). To mimic influenza infections, we infected BMDCs with different MOIs of PR8 viruses and treated them with rIL-33. At 24 h post-infection, we harvested cell culture media and measured levels of IL-12p40 levels. The higher MOIs induced a larger production of IL-12p40 by BMDCs, and rIL-33 treatment amplified these productions (Figure 3C). In addition, we used flow cytometry to analyze the expression of CD86 and MHC class II (MHCII) in BMDCs at 24 h post-rIL-33 treatment. Treatment with rIL-33 increased the expression of CD86 and MHCII in BMDCs (Figure 3D). Overall, exogenous IL-33 enhanced the secretion of IL-12p40 in BAL fluids post-infection, amplified IL-12 production, and increased the maturation of BMDCs.

### 3.4. Exogenous IL-33, but Not Endogenous IL-33, Enhances Cytotoxic T-Cell (CTL) Responses against Influenza Virus

**Figure 4:**
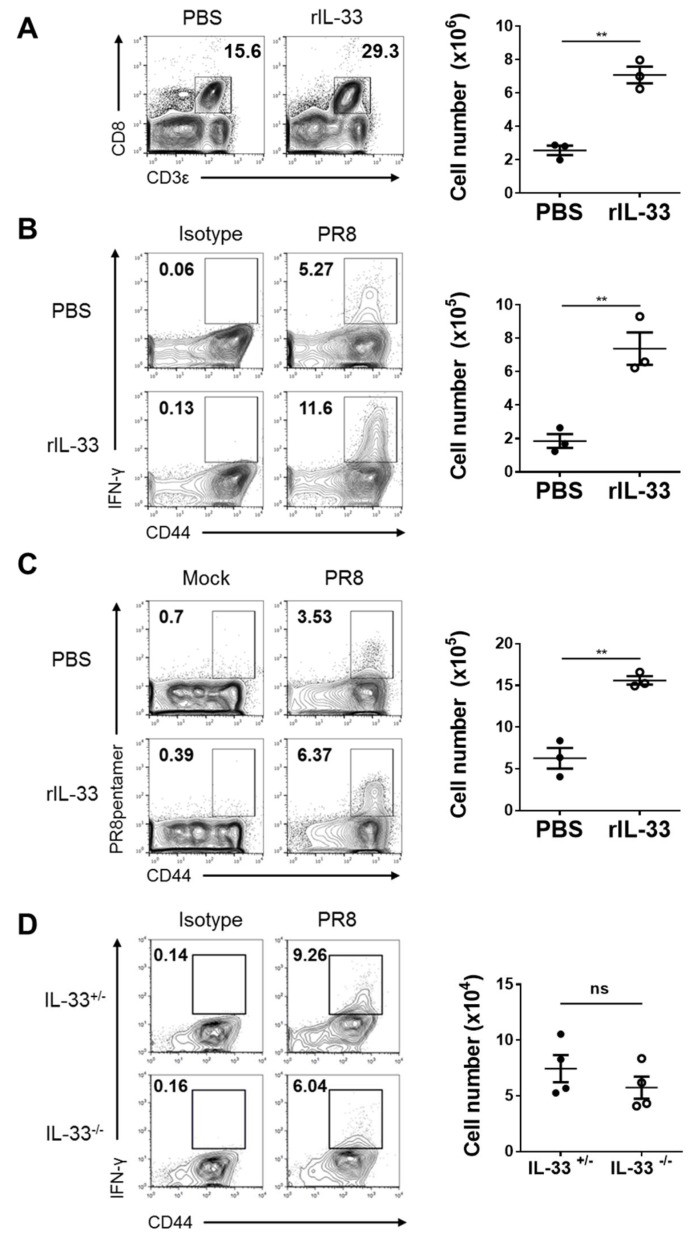
Exogenous IL-33, but not endogenous IL-33, enhanced CD8 T-cell responses against influenza infection. (**A**–**C**) C57BL/6 mice were injected intranasally with 0.5 μg of rIL-33 or PBS daily for five days and infected with 50 PFU of PR8 influenza virus. (**A**) On day seven post-infection, the recruitment of CD3ε^+^ CD8^+^ T-cells to lungs was assessed by flow cytometry. (**B**) IFN-γ production by CD3ε^+^ CD8^+^ CD44^hi^ T-cells post-stimulation with NP_366–374_ peptide was measured by intracellular staining. (**C**) NP_366-374_ antigen-specific CD3ε^+^ CD8^+^ CD44^+^ T-cells were measured by pentamer staining. (**D**) IL-33^+/−^ and IL-33^−/−^ mice were infected with 50 PFU of PR8 influenza virus. On day seven post-infection, IFN-γ production by CD3ε^+^ CD8^+^ CD44^hi^ T-cells post-stimulation with NP_366-374_ peptide was measured by intracellular staining. ** *p* < 0.01; ns, not significant.

For host defense from influenza infection, it is important that innate immune responses rapidly occur. However, adaptive immune responses are also essential. In particular, cytotoxic T-cells (CTLs) play a critical role in antigen specificity and cytokine production [25,26]. Thus, we investigated whether exogenous IL-33 affects CTL responses against influenza infection. At seven days post-infection, we isolated cells from lungs injected with exogenous IL-33 daily for five days prior to infection. The number of CD8^+^ T-cells in exogenous IL-33-injected lungs were significantly increased, as assessed via flow cytometry (Figure 4A). In addition, exogenous IL-33 increased the number of CD8^+^ T-cells that produced IFN-γ (Figure 4B) or were specific to the PR8 virus antigen (Figure 4C). On days three and five post-infection, there were no significant differences in the number of IFN-γ-producing CD8^+^ T-cells (Figure S2).

Next, to confirm whether endogenous IL-33 influenced CTL responses against influenza infection, IL-33^+/−^ and IL-33^−/−^ mice were infected with the influenza virus. Cells were then isolated from lungs at seven days post-infection and analyzed by flow cytometry. Unlike exogenous IL-33, endogenous IL-33 deficiency did not affect the number of IFN-γ-producing CD8^+^ T-cells (Figure 4D). These results demonstrate that exogenous IL-33 enhanced the specificity to the influenza virus and the CTL production of CD8^+^ T-cells. In contrast, endogenous IL-33 had no effect.

## 4. Discussion

Our findings suggest an antiviral effect of exogenous IL-33 in the lung against influenza infection. Local administration of exogenous IL-33 reinforced antiviral immunity, resulting in increased survival and viral clearance. However, the deficiency of endogenous IL-33 did not affect mortality post-infection. Exogenous IL-33 induced immune cell recruitment into the lung; these cells included ILC2s, eosinophils, and DCs. However, alveolar macrophages were decreased post-treatment. Additionally, exogenous IL-33 facilitated the in vivo secretion of IL-12, which is an important pro-inflammatory cytokine in innate and adaptive immunity. Furthermore, the in vitro treatment of exogenous IL-33 induced the production of IL-12 by BMDCs and enhanced BMDC maturation with increased levels of CD86 and MHCII. Finally, exogenous IL-33 enhanced CTL response against influenza infection, which resulted in an increase in IFN-γ production and the PR8 specificity of CD8^+^ T-cells post-viral infection. Endogenous IL-33 had no effect on the abovementioned responses. These results suggest that exogenous IL-33 contributes to the enhanced antiviral immunity against influenza infection.

IL-33 is a cytokine that is produced and released upon influenza infection [7]. Previous studies have shown that IL-33-responding ILC2s occur in influenza virus-induced hyper-reactivity and present as asthma-like symptoms [13]. However, we found that the level of IL-33 in BAL fluids was much less than that of IL-6 and IL-12p40, and the survival of IL-33-deficient mice was similar to those of normal mice post-infection. This suggests that the asthma-like symptoms induced by influenza infection do not greatly affect mortality against viral infection in our experimental model, due to minimal IL-33 secretion. Furthermore, we observed that the injection of exogenous IL-33 prior to viral infection increased the survival of influenza-infected mice and induced ILC2 recruitment into the lung. Other studies have also shown that in response to IL-33, ILC2s produce amphiregulin, thus leading to the restoration of lung tissues infected with influenza virus [14]. Thus, it is likely that exogenous IL-33 might stimulate ILC2s to contribute to virus-infected lung tissue restoration, rather than exacerbation.

In our study, the intranasal injection of rIL-33 (0.5 μg/mouse) daily for five days increased IL-12p40 levels in BAL fluids and recruited DCs, but not alveolar macrophages, into the local microenvironment. In contrast, a previous study showed that intranasal injections with larger amounts of rIL-33 (4 μg/mouse) for six consecutive days induced the recruitment of macrophages into the lung, which led to the polarization of alternatively activated macrophages for type 2 inflammation [27]. These two different outcomes might be attributed to differences in the amount of injected rIL-33. Additionally, we found that IL-33 treatment induced BMDCs to generate a larger amount of IL-12p40 and to express higher levels of MHCII and CD86, which function predominantly in antigen presentation. These results suggest that the increase in DCs by a moderate amount of exogenous IL-33 may be a source of IL-12 in the infection site and a main player of antigen presentation, leading to the initiation of the CTL response against influenza infection.

Though neutralizing antibodies are a highly effective defense immunity, CTLs still play an important role in situations in which the virus evades antibodies and infects epithelial cells [3]. CTLs are able to kill the infected cell before viral progenies spread to surroundings, resulting in viral clearance [28]. Our results show that exogenous IL-33, but not endogenous IL-33, increased the recruitment of CD8^+^ T-cells into lungs following influenza infection. Additionally, exogenous IL-33 facilitated the production of IFN-γ and the specificity of CD8^+^ T-cells to the PR8 virus on day seven. This effect was not observed at the early time points, suggesting that IL-33 enhances the status of CD8^+^ T-cells rather than their response rate. A previous study showed that the in vitro treatment of IL-33 promotes CTL responses with the stimulation of IL-12 and T-cell receptor signaling [29]. Overall, these findings suggest that elevated IL-12p40 levels and DC recruitment by exogenous IL-33 injection prior to viral infection can enhance CTL responses against the influenza virus.

Previous studies have shown low but significantly elevated levels of serum IL-33 in patients with seasonal influenza A [30]. In contrast, higher levels of IL-33 were measured in asthmatic patients, and these levels increased with the severity of the disease, suggesting that elevated IL-33 is responsible for the exacerbation of asthmatic diseases [31]. IL-33 also regulates other allergic diseases, including atopic dermatitis [32]. Due to the likelihood of side effects with these allergic diseases, there is a paucity of research on the potential therapeutic role of IL-33 in humans.

In contrast, the therapeutic potential of IL-33 has been suggested in several studies on mouse models of human disease. In coxsackie B virus-induced pancreatitis, the administration of IL-33 enhances CD8^+^ T-cells and the IFN-γ-dependent antiviral immune response, resulting in a decrease in viral loads and the attenuation of pancreatitis [33]. In the human papilloma virus-associated cancer model, IL-33 increases antigen-specific CD8^+^ T-cell responses and induces tumor regression [34]. Furthermore, Tsunoda and colleagues reported that IL-33 is able to act as a mucosal vaccine adjuvant by enhancing protective immune responses against influenza infection [35]. Along with these studies, our findings might support the potential of IL-33 as a therapeutic drug.

In conclusion, our findings demonstrate that exogenous IL-33 enhanced antiviral immune responses against respiratory influenza infection by inducing the activation of DCs and antigen-specific CD8^+^ T-cell responses. Results from this study also suggest that IL-33 may be a candidate of treatments for mucosal influenza viral infection.

## Supplementary Materials

Supplementary materials can be found at https://www.mdpi.com/1999-4915/11/9/840/s1.
