# Supplementary material for: Exogenous Interleukin-33 Contributes to Protective Immunity via Cytotoxic T-Cell Priming against Mucosal Influenza Viral Infection
Source: Viruses. 2019 Sep 10;11(9):840. doi: 10.3390/v11090840 (PMC6783873; doi:10.3390/v11090840)
Supplement: Supplementary file 1 [file viruses-11-00840-s001.pdf]

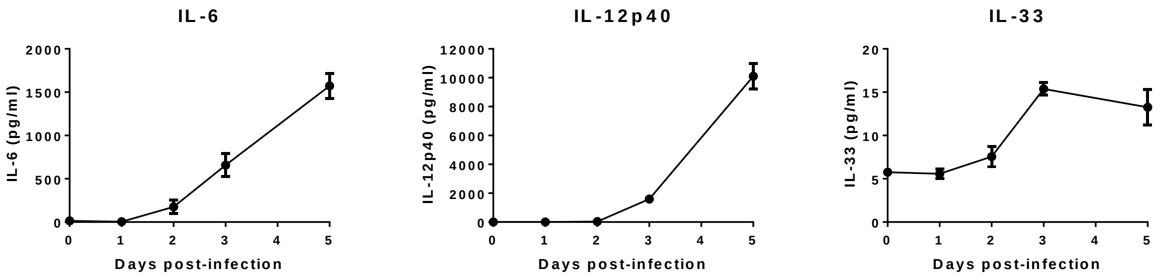

Figure S1. Levels of interleukin (IL)-6, IL-12p40, and IL-33 in bronchoalveolar lavage (BAL) fluids post-infection.

C57BL/6 mice (n = 3 per group) were infected intranasally with 50 PFU of PR8 influenza virus. At the indicated days post-infection, BAL fluids were collected, and the levels of IL-6, IL-12p40, and IL-33 in BAL fluids were measured by enzyme-linked immunosorbent assay (ELISA).

Kim et al. Supplementary Figure 1

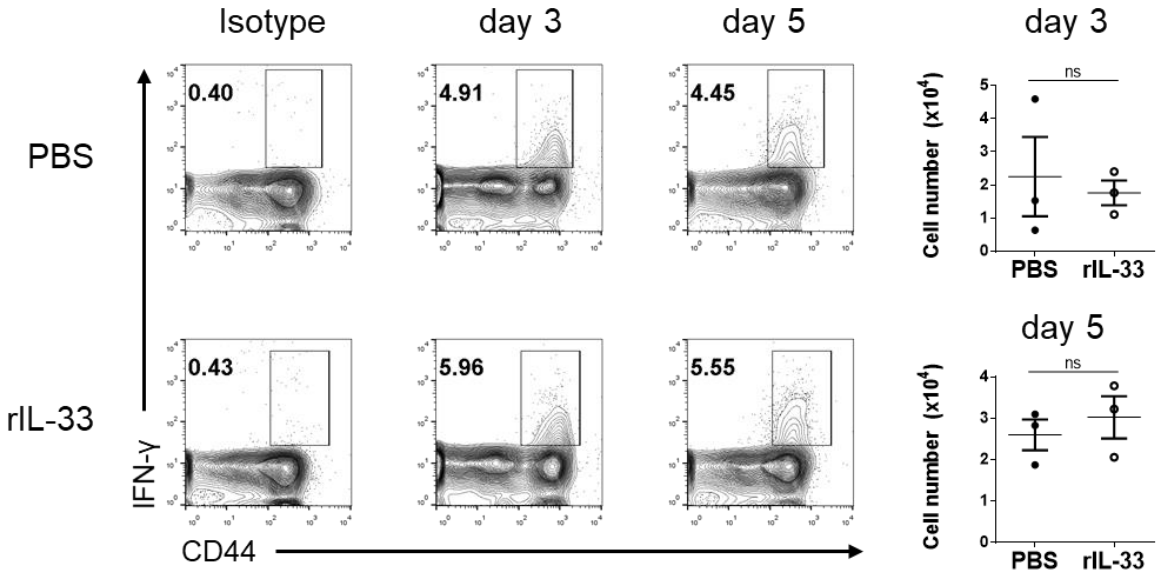

Figure S2. The number of IFN-γ-producing CD8$^+$ T cells in the lungs 3 and 5 days post-infection. C57BL/6 mice (n = 3 per group) were injected intranasally with 0.5 μg rIL-33 or phosphate-buffered saline (PBS) daily for 5 days and infected with 50 PFU of PR8 influenza virus. On days 3 and 5 post-infection, IFN-γ production by CD3ε$^+$ CD8$^+$ CD44^{hi} T cells following stimulation with PMA and ionomycin was mea sured by intracellular staining. ns, not significant.
